# Cellulose paper support with dual-layered nano–microstructures for enhanced plasmonic photothermal heating and solar vapor generation†

**DOI:** 10.1039/d0na00163e

**Published:** 2020-04-22

**Authors:** Yintong Huang, Yoshitaka Morishita, Kojiro Uetani, Masaya Nogi, Hirotaka Koga

**Affiliations:** The Institute of Scientific and Industrial Research, Osaka University 8-1 Mihogaoka Ibaraki Osaka 567-0047 Japan hkoga@eco.sanken.osaka-u.ac.jp +81-6-6879-8444 +81-6-6879-8442

## Abstract

Plasmonic nanoparticles, such as gold nanoparticles (AuNPs), have been actively applied in solar vapor generation for seawater desalination and water purification, owing to their photothermal heating performances. Such nanoparticles have been frequently anchored within porous supporting materials to ensure easy handling and water absorption. However, there has been limited progress in improving the transport efficiency of light to nanoparticles within porous supports to achieve more effective photothermal heating. Here, we show an enhanced light absorption of AuNPs by supporting on a cellulose paper with tailored porous structures for efficient photothermal heating. The paper consists of AuNP-anchored cellulose nanofibers and cellulose pulp as the top and bottom layers, respectively, which provides dual-layered porous nano–microstructures in the perpendicular direction. Then, the bottom layer with pulp-derived microstructures reflects the transmitted light back to AuNPs within the top layer, which improves their light absorptivity. Thus, under 1 sun illumination, the dual-layered paper demonstrates superior performance in photothermal heating (increases from 28 °C to 46 °C) and solar vapor generation (1.72 kg m^−2^ h^−1^) compared with the single-layered AuNP-anchored cellulose nanofiber paper even at the same AuNP content. Furthermore, the water evaporation rate per AuNP content of the dual-layered paper is more than 2 times higher than those of the state-of-the-art AuNP-anchored porous materials under the same light irradiation. This strategy enables the efficient use of precious plasmonic nanoparticles for further development of solar vapor generation.

## Abstract 2

Light absorption and photothermal heating performance of plasmonic nanoparticles are enhanced by a cellulose paper support with dual-layered nano/microstructures of cellulose nanofibers and pulp fibers for efficient solar vapor generation.

## Graphical abstract

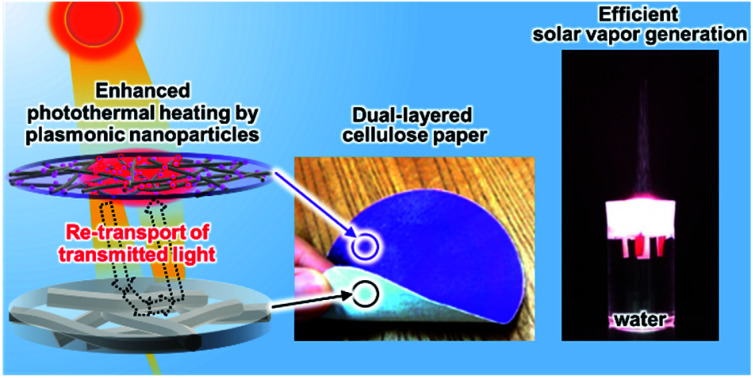

## 1. Introduction

Plasmonic nanoparticles, such as gold nanoparticles (AuNPs), have a photothermal heating ability with which they can absorb light energy and convert it to thermal energy, owing to their localized surface plasmon resonance effect.^1,2^ Recently, solar light-driven photothermal heating by AuNPs has been applied to vaporize water for seawater desalination and water purification.^3–6^ In the primary studies of the solar vapor generation, AuNPs were suspended in water and then irradiated by solar light. However, the photothermal heating performance of AuNPs inevitably deteriorated owing to the aggregation of AuNPs in the water suspension. In addition, an excess thermal energy was required to heat the bulk water around the AuNPs, which resulted in a low efficiency of the solar vapor generation process.^7^

To overcome the aggregation of AuNPs in a water suspension and the heat loss to bulk water, many efforts have been made to anchor AuNPs within porous supporting materials, such as cellulose paper,^8–10^ air-laid paper,^11,12^ synthetic polymer nanofibrous films^13^ and nanoporous alumina.^14^ When an AuNP-anchored porous material was floated on water and then irradiated by solar light from its upper surface, the thermal energy generated from the AuNPs enabled the selective heating of the water surface, which improved the water evaporation rate by the suppression of heat loss.^10^ Among the various kinds of porous supports, paper materials, which are prepared from wood-derived cellulose fibers, are promising for the solar vapor generation process, owing to their abundance, sustainability, thermal insulating property, and hydrophilic and water-absorbing properties.^15^

The recent research into improving the performance of AuNP-mediated solar vapor generation has focused on the abundant use of AuNPs,^6^ the design of the size^16^ and shape^9,17^ of AuNPs, anchoring AuNPs on porous supports,^8–14,18^ or the design of the evaporation system setup.^19^ Because Au is an expensive precious metal, its efficient use is an urgent issue. Therefore, it is essential to take full advantages of the photothermal heating ability of AuNPs without using a large number of AuNPs. From this viewpoint, there is a strong requirement to improve the light absorption efficiency of AuNPs anchored within porous supports. Tailoring the porous structures of supporting materials is expected as one of the most promising approaches to realize efficient light transport to the anchored AuNPs. However, there has been limited progress in porous-structure design of the supporting material to enhance the light absorption of AuNPs for effective photothermal heating and solar vapor generation.

**Fig. 1:**
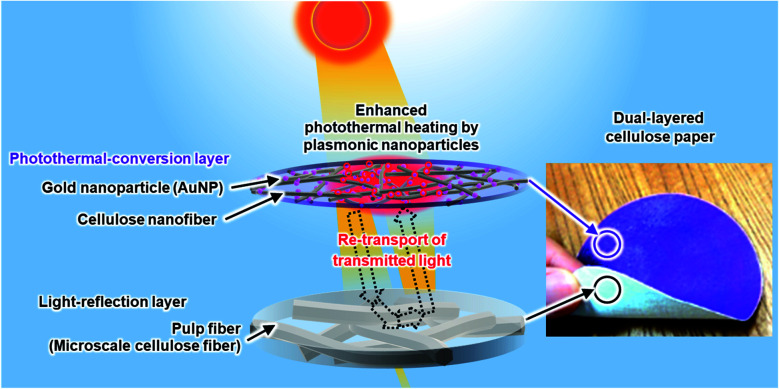
Schematic illustration of the solar-driven photothermal heating by AuNP@cellulose paper with dual-layered nano/micro porous structures consisting of AuNP@cellulose nanofibers and pulp fibers as top (photothermal-conversion) and bottom (light-reflection) layer, respectively.

Here we show the AuNP-anchored porous paper support with tailored cellulose-fiber nano/microstructures for enhanced photothermal heating and solar vapor generation. The paper support consists of the photothermal-conversion layer (upper layer) of AuNP-anchored cellulose nanofibers (AuNP@cellulose nanofibers) and the light-reflection layer (bottom layer) of AuNP-free cellulose pulp fibers (Fig. 1). This dual-layered nano/micro porous structure improves the light absorption efficiency of the anchored AuNPs, which leads to effective photothermal heating and solar vapor generation; the water evaporation rate per AuNP content is more than 2 times higher than those of the state-of-the-art AuNP-anchored porous materials under the same solar light irradiation.

## 2. Results and discussion

### 2.1 Photothermal heating performance of AuNP@cellulose nanofiber paper

**Fig. 2:**
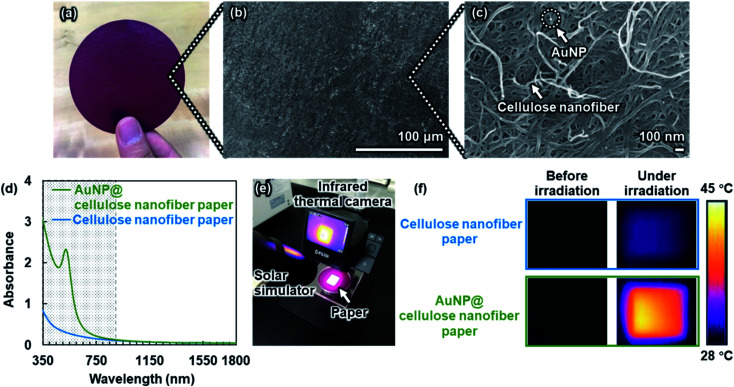
(a) Optical and (b and c) SEM images of the AuNP@cellulose nanofiber paper, (d) UV-vis-NIR absorbance spectra of the AuNP@cellulose nanofiber paper and the cellulose nanofiber paper, (e) optical image of the experiment setup for surface temperature measurement during simulated solar light irradiation, and (f) infrared images of the cellulose nanofiber paper (thickness: *ca.* 80 μm, emissivity: 0.87) and the AuNP@cellulose nanofiber paper (thickness: *ca.* 100 μm, AuNP content: 3.3 mmol m^−2^, emissivity: 0.90) before and under 1.0 kW m^−2^ (1 sun) irradiation.

Cellulose nanofibers, which are obtained by nanofibrillation of wood-derived cellulose pulp fibers, can serve as effective support materials for AuNPs, owing to their large specific surface areas.^20,21^ In this study, the AuNP@cellulose nanofibers were fabricated into the paper with nanoporous structures, and its photothermal heating performance was then evaluated. The preparation of the AuNP@cellulose nanofiber paper was performed according to our previous report.^21^ Briefly, an aqueous suspension of softwood-derived cellulose nanofibers (widths: 5–50 nm), with weakly negative charge, was mixed with an aqueous solution of polyethyleneimine (PEI) containing a high-density positive charge to afford positively charged cellulose nanofibers. Negatively charged Au precursor ions ([AuCl_4_]^−^) were then attached to the positively charged cellulose nanofibers through electrostatic interaction in the suspension, followed by suction filtration. Subsequently, the solvent exchange treatment with *tert*-butyl alcohol (*tert*-BuOH) and hot-press drying was conducted to form the AuNP@cellulose nanofiber paper with nanoporous structures (diameter: 75 mm, thickness: *ca.* 90 μm, AuNP content: 3.3 mmol m^−2^) (Fig. 2a–c, see also Fig. S1†). Then, the AuNPs were formed *in situ* on the cellulose nanofibers by PEI as reductant for Au precursor ions ([AuCl_4_]^−^).^21^ The as-prepared AuNP@cellulose nanofiber paper was transparent and had a red-purple color, and showed absorption of light with wavelengths below 900 nm, owing to the specific light absorption phenomenon arising from the localized surface plasmon resonance effect of AuNPs (Fig. 2d).^22,23^ Then, the photothermal heating performance of the AuNP@cellulose nanofiber paper was evaluated by irradiation of the simulated solar light (AM1.5G, wavelength: 350–1800 nm, light intensity: 1.0 kW m^−2^ (1 sun)). The change in temperature of the paper samples under solar light irradiation was measured using an infrared thermal camera, which was corrected by their actual emissivity. As shown in Fig. 2e and f, the temperature of the AuNP@cellulose nanofiber paper increased by 14.5 ± 0.27 °C (from 28 to 42.5 °C), while that of the AuNP-free cellulose nanofiber paper increased by only 2 °C under the same irradiation conditions (see also Fig. S2†). This higher temperature increase suggested that the plasmonic AuNP anchored within the cellulose nanofiber paper support allowed for effective photothermal heating.

### 2.2 Enhanced photothermal heating by the structure design of cellulose paper support

**Fig. 3:**
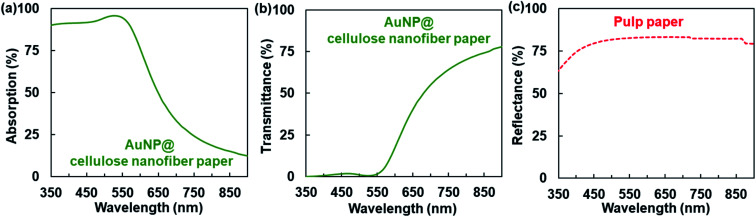
(a) UV-vis-NIR absorption and (b) transmittance spectra of the AuNP@cellulose nanofiber paper (thickness: *ca.* 100 μm, AuNP content: 3.3 mmol m^−2^), and (c) reflectance spectrum of the AuNP-free pulp paper (thickness: *ca.* 410 μm, pulp content: *ca.* 800 mg).

As described above, the AuNP@cellulose nanofiber paper provided the temperature to increase by 14.5 ± 0.27 °C under simulated solar light irradiation (wavelength: 350 to 1800 nm, light intensity: 1.0 kW m^−2^ (1 sun)), because the AuNPs within the cellulose nanofiber paper absorbed light energy in the wavelength range of 350–900 nm (Fig. 2d) and converted it into thermal energy. Efficient light absorption of AuNPs is crucial to achieve effective photothermal heating. However, the AuNP@cellulose nanofiber paper indicated an insufficient light absorption; the light absorption was drastically decreased at wavelengths above 550 nm (Fig. 3a). Although an increase of the AuNP content is considered to be effective to improve the light absorption, the use of an excess amount of expensive and precious Au is unfavorable.

For this reason, we then investigated a way to improve the light absorption efficiency of AuNPs by tailoring porous structures of the paper support. The AuNP@cellulose nanofiber paper showed a high total light transmittance in the wavelength range of 550–900 nm (Fig. 3b), where the light absorption of AuNPs was decreased (Fig. 3a). When the unused light energy, which is transmitted through the transparent AuNP@cellulose nanofiber paper without being absorbed by the AuNPs, can be re-transported to the AuNPs, their light absorption efficiency would be improved. Herein, we focused on the traditional white paper made from cellulose pulp fibers with microscale widths, which can reflect light owing to its porous microstructures of pulp fiber networks. In actuality, it was confirmed that the pulp paper showed high reflectance over 75% in the wavelength range of 350–900 nm (Fig. 3c). The reflection of the transmitted light through the AuNP@cellulose nanofiber paper by the pulp paper would be effective for the re-transport of light to the AuNPs. Thus, integrating the AuNP@cellulose nanofiber paper with the pulp paper is expected as a promising way to enhance light absorption and the photothermal heating performance (Fig. 1).

**Fig. 4:**
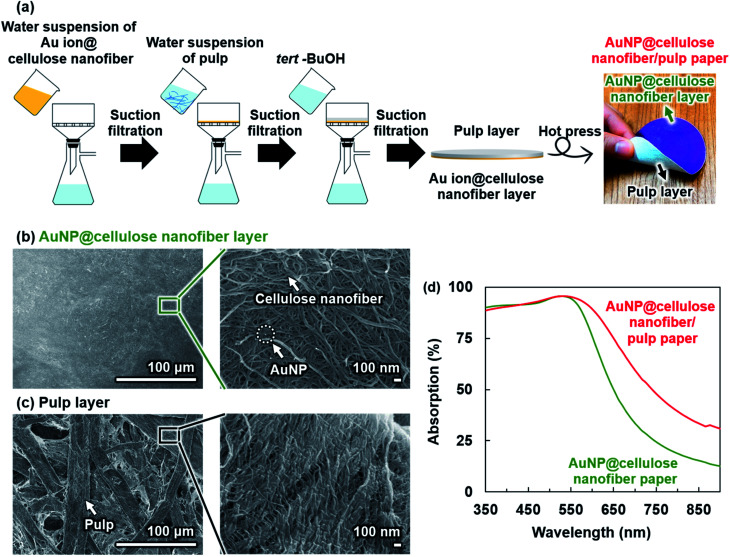
(a) Preparation procedure of the AuNP@cellulose nanofiber/pulp paper, FE-SEM images of (b) the AuNP@cellulose nanofiber layer and (c) the pulp layer of the AuNP@cellulose nanofiber/pulp paper, and (d) UV-vis-NIR absorption spectra of the AuNP@cellulose nanofiber/pulp paper (thickness: *ca.* 520 μm, AuNP content: 3.3 mmol m^−2^, pulp content: *ca.* 800 mg) and the AuNP@cellulose nanofiber paper (thickness: *ca.* 100 μm, AuNP content: 3.3 mmol m^−2^).

To prove this concept, the paper with dual-layered structures of AuNP@cellulose nanofibers and AuNP-free pulp fibers was prepared by a two-step papermaking process (Fig. 4a). This sequential procedure allowed the deposition of the AuNP free-pulp fiber layer to the surfaces of the AuNP@cellulose nanofiber paper without changing the Au loading, which was quantitatively confirmed by an atomic absorption analysis. The as-prepared paper (thickness: *ca.* 520 μm, Au loading: 3.3 mmol m^−2^, pulp content: *ca.* 800 mg), denoted as the AuNP@cellulose nanofiber/pulp paper, had the AuNP@cellulose nanofiber-network porous nanostructures in the top layer and the pulp-network porous microstructures in the bottom layer (Fig. 4b and c), while keeping paper-specific flexibility for easy handling (Fig. 4a). There would be also no large difference of the size and dispersion state of the AuNPs between the AuNP@cellulose nanofiber papers with and without the pulp layer. This is owing to the preparation procedure, where the AuNP@cellulose nanofiber/pulp paper was prepared by using a water suspension of the pulp fiber (200 mL), instead of the distilled water (200 mL) in the case of the AuNP@cellulose nanofiber paper. Then, the AuNP@cellulose nanofiber/pulp paper demonstrated an improvement of light absorption in the wavelength range of 550–900 nm compared with the AuNP@cellulose nanofiber paper without the pulp layer (Fig. 4d, see also Fig. S3 and S4†). Both the reflectance of the pulp paper and the light absorption of the AuNP@cellulose nanofiber/pulp paper were increased by increasing the pulp content (thickness) (Fig. S5†). Therefore, the integration of the AuNP@cellulose nanofibers with the pulp fibers as a light-reflection layer could enhance the light absorption efficiency of the AuNPs, suggesting that the pulp layer-mediated reflection of the transmitted light through the AuNP@cellulose nanofiber paper provided the re-transport of light to the AuNPs.

**Fig. 5:**
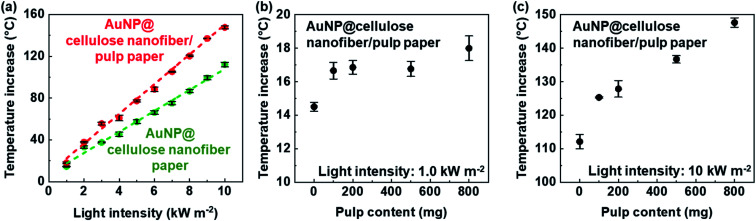
(a) Temperature increase *versus* solar light intensity of the AuNP@cellulose nanofiber paper (AuNP content: 3.3 mmol m^−2^, emissivity: 0.90) and the AuNP@cellulose nanofiber/pulp paper (AuNP content: 3.3 mmol m^−2^, pulp content: *ca.* 800 mg, emissivity: 0.90), and temperature increase *versus* pulp content of the AuNP@cellulose nanofiber/pulp paper (AuNP content: 3.3 mmol m^−2^, emissivity: 0.90) under (b) 1.0 kW m^−2^ and (c) 10 kW m^−2^ irradiation.

The photothermal heating performance was then evaluated. When the AuNP@cellulose nanofiber/pulp paper was irradiated by simulated solar light (light intensity: 1.0 kW m^−2^ (1 sun)) from the side of the AuNP@cellulose nanofiber layer, its temperature increased by 18.0 ± 0.74 °C (from 28 to 46 °C), which was 3.5 °C higher than the AuNP@cellulose nanofiber paper without the pulp layer (14.5 ± 0.27 °C) (Fig. S6†). This result suggested that the pulp layer improved the photothermal heating performance. The effect of the pulp layer increased with increasing the light intensity; the temperature increase of the AuNP@cellulose nanofiber/pulp paper was approximately 50 °C higher than that of the AuNP@cellulose nanofiber paper without the pulp layer at the light intensity of 10 kW m^−2^ (10 sun) (Fig. 5a). It was also confirmed that the temperature increase was improved with increasing the pulp content (Fig. 5b and c) owing to the enhanced light absorption efficiency of the AuNPs by the pulp layer (Fig. S6†). Thus, the tailored structures consisting of the AuNP@cellulose nanofibers as a photothermal conversion layer and the pulp fibers as a light reflection layer successfully enhanced both the light absorption efficiency and the photothermal heating performance without needing to increase the AuNP content.

### 2.3 Applications for solar vapor generation and seawater desalination

**Fig. 6:**
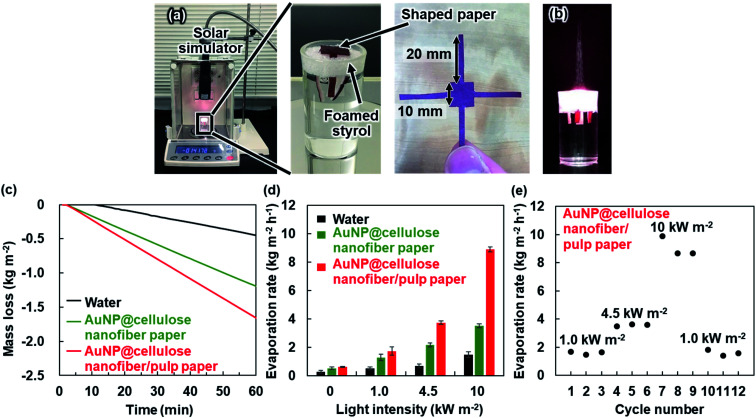
Optical images of (a) the experimental setup for evaluating mass loss during the solar vapor generation process and (b) the solar vapor generation under simulated solar light illumination, (c) mass loss arising from evaporation under 1.0 kW m^−2^ irradiation over time, and (d) the evaporation rate under 0, 1.0, 4.5 and 10 kW m^−2^ irradiation for water (without paper), the AuNP@cellulose nanofiber paper (AuNP content: 3.3 mmol m^−2^) and the AuNP@cellulose nanofiber/pulp paper (AuNP content: 3.3 mmol m^−2^, pulp content: *ca.* 800 mg), (e) the repeated seawater-evaporation performance of the AuNP@cellulose nanofiber/pulp paper (AuNP content: 3.3 mmol m^−2^, pulp content: *ca.* 800 mg) under 1.0, 4.5 and 10 kW m^−2^ irradiation.

The high photothermal heating performance of the AuNP@cellulose nanofiber paper was applied for the solar vapor generation process. Fig. 6a and b displays the experimental setup. The AuNP@cellulose nanofiber paper or the AuNP@cellulose nanofiber/pulp paper was cut into a certain shape, and then placed on foamed styrol floating on the surface of distilled water. Then, the mass loss of the water under simulated solar light irradiation of the paper was monitored. When the distilled water was irradiated by 1.0 kW m^−2^ (1 sun) simulated solar light, water vapor was generated owing to evaporation, and a mass loss of 0.52 kg m^−2^ was observed after 1 h. When the AuNP@cellulose nanofiber paper was used, the mass loss of water increased by 1.27 kg m^−2^, which indicated that the photothermal heating by AuNP allowed effective water vapor generation. The AuNP@cellulose nanofiber/pulp paper further increased the mass loss up to 1.72 kg m^−2^ (Fig. 6c). As shown in Fig. 6d, the AuNP@cellulose nanofiber/pulp paper achieved a higher water evaporation rate than the AuNP@cellulose nanofiber paper even at the same AuNP content. The higher evaporation rate of the AuNP@cellulose nanofiber/pulp paper was clearer at higher light intensities (Fig. 6d), owing to the enhanced photothermal heating performance of the AuNP by the pulp layer (Fig. 5a). From these results, it was confirmed that the dual-layered AuNP@cellulose nanofiber/pulp paper with high photothermal heating performance can also achieve an efficient evaporation rate compared with the single-layered AuNP@cellulose nanofiber paper without the pulp layer. The AuNP@cellulose nanofiber/pulp paper demonstrated a higher evaporation rate per the AuNP content, as compared with those of the state-of-the-art AuNP-based materials under the similar solar light intensities (Table 1).

**Table 1:** Solar vapor generation performances of the state-of-the-art AuNP-based materials

Entry	Materials	Light intensity [kW m^−2^]	Au content [mmol m^−2^]	Evaporation rate [kg h^−1^ m^−2^]	Evaporation rate/Au content [kg h^−1^ mmol^−1^]	Ref.
1	A self-assembled film of AuNP	50.9	4.0^a^	36.67^b^	9.17	24
2	Airlaid-paper-based AuNP film	4.5	7.9^a^	5.00^b^	0.63	11
3	AuNP/poly(*p*-phenylene benzobisoxazole) nanofiber composite	1.0	19.4^a^	1.424	0.07	13
4	AuNP film on filter paper	10	14.5^a^	11.80	0.81	10
		1.0	14.5^a^	0.87	0.06	10
5	AuNP@cellulose nanofiber/pulp paper	10	3.3	8.89	2.69	This work
		4.5	3.3	3.73	1.13	This work
		1.0	3.3	1.72	0.52	This work

a Au content values, which were calculated from the data given in the corresponding references.

b Evaporation rate values, which were calculated from the data given in the corresponding references.

Finally, seawater-evaporation testing was carried out by using artificial seawater for seawater desalination. The seawater evaporation rate of the AuNP@cellulose nanofiber/pulp paper was 1.59 kg m^−2^ h^−1^ at a light intensity of 1.0 kW m^−2^ (1 sun), which was almost the same when using distilled water. This performance could be maintained even after long-time and repeated use (Fig. 6e). Thus, the AuNP@cellulose nanofiber/pulp paper is expected to be a promising material for solar vapor generation with efficient use of AuNPs.

## 3. Conclusion

In conclusion, the AuNP@cellulose nanofiber/pulp paper with dual-layered nano/micro porous structures was developed by integrating the AuNP@cellulose nanofiber and the pulp fiber for solar-driven photothermal heating and the vapor generation process. Design of the dual-layered structures within the paper support could enhance the light absorption and photothermal heating performance of the anchored-AuNPs, which also led to the improvement of the solar vapor generation performance. The AuNP@cellulose nanofiber/pulp paper described here demonstrated a higher evaporation rate per AuNP content compared with the previously-reported AuNP-based materials, which contributes to the effective use of expensive precious Au metal. This tailored paper material could also be applied to solar-mediated seawater desalination with high reusability. This concept, which is for improving the photothermal heating performance of AuNPs by designing the porous structures within the paper support, can be applied to various photothermal materials, which will open new doors for the solar vapor generation process.

## 4. Experimental

### 4.1 Materials

Cellulose nanofibers were prepared from never-dried softwood bleached kraft pulp according to the modified method of our previous report.^25^ An aqueous suspension of the pulp (0.2 wt%, 2 L) was treated by a high-pressure water-jet system (Star Burst, HJP-25008, Sugino Machine Co., Ltd, Uozu, Japan) equipped with a counter-collision chamber. The pulp suspension was ejected from a nozzle with a diameter of 0.10 mm at 245 MPa with 100 cycle repetitions. PEI (average molecular weight: 1800 Da) was purchased from Wako Pure Chemical Industries, Ltd., Osaka, Japan. *tert*-BuOH (>99.0% purity) was obtained from Tokyo Chemical Industry Co., Ltd., Tokyo, Japan. HAuCl_4_·3H_2_O (98% purity) was purchased from Sigma-Aldrich Japan, Ltd., Tokyo, Japan. Artificial seawater (Marine Art SF-1) was purchased from Tomita Pharmaceutical Co., Ltd., Tokushima, Japan. All chemicals were used as received without any further purification.

### 4.2 Preparation of AuNP@cellulose nanofiber paper and AuNP@cellulose nanofiber/pulp paper

The AuNP@cellulose nanofiber paper was prepared similar to our previous report.^21^ An aqueous suspension of the cellulose nanofiber (0.075 wt%, 200 mL) was mixed with an aqueous solution of PEI (1.0 wt%, 0.2 mL) and 10 mL of the aqueous solution of HAuCl_4_ (1 g L^−1^) in that order, with each step being carried out at intervals of 15 min. The obtained suspension was dewatered by suction filtration through a membrane filter (H020A090C, hydrophilic polytetrafluoroethylene (PTFE) membrane, 0.2 μm pore diameter, Advantec Toyo Roshi Kaisha, Ltd., Tokyo, Japan). Distilled water (200 mL) and the *tert*-BuOH (300 mL) were then poured in, in that order, and gently filtered. The resulting wet sheet on the membrane filter was sandwiched between a membrane filter and paper towels, and was then treated by hot pressing at 110 °C for 30 min (1 MPa). The obtained sample (*Φ* 75 mm) was peeled from the membrane filter to prepare the AuNP@cellulose nanofiber paper. The cellulose nanofiber paper was prepared without adding the aqueous solution of HAuCl_4_. For preparation of the AuNP@cellulose nanofiber/pulp paper, an aqueous suspension of the pulp fiber (200 mL) with a specified concentration (0.05, 0.1, 0.25, 0.4 wt%) was used, instead of the distilled water (200 mL).

### 4.3 Analyses

The porous structure of AuNP@cellulose nanofiber paper and AuNP@cellulose nanofiber/pulp paper were observed by a field-emission scanning electron microscope (FE-SEM, SU-8020, Hitachi High-Tech Science Corp., Tokyo, Japan). The AuNP contents were determined by an atomic absorption spectrophotometer (ZA3300, Hitachi High-Tech Science Corp., Tokyo, Japan). Transmittance, reflectance and absorbance spectra were measured by a UV-vis-NIR spectrophotometer (UV-3600 Plus, Shimadzu Corp., Kyoto, Japan) with an integrating sphere attachment (ISR-603, Shimadzu Corp., Kyoto, Japan). Absorption spectra were calculated from the transmittance and reflectance spectra as shown in Fig. S3.† The X-ray diffraction (XRD) patterns were recorded by an Ultima IV (Rigaku Corp., Tokyo, Japan), with a scanning angle (2*θ*) range of 5–80°. A digital thickness gauge (G2N-255M, Ozaki MFG. Co., Ltd., Tokyo, Japan) was used for the measurement of the thickness.

### 4.4 Surface temperature measurement during solar light illumination

Before the temperature measurement, the emissivity of each sample was measured. A black tape (HB-250, OPTEX Co., Ltd., Shiga, Japan) with a known emissivity of 0.95 was used as a reference for the emissivity measurement. The paper and the black tape were heated to 50 °C by a temperature controller (SBX-303, Sakaguchi E.H VOC Corp., Tokyo, Japan). Then, a digital thermometer (MT-309, MotherTool Co. Ltd., Ueda, Japan) with a thermal couple sensor (TP-11, MotherTool Co. Ltd., Ueda, Japan) was used to ensure that both the paper and the black tape achieved the same temperature. By adjusting the temperature of the paper measured with an infrared thermal camera (FLIR ETS320, FLIR Systems. Inc., Wilsonville, USA), according to the reference temperature of the black tape, the emissivity of the paper was measured. In the temperature measurement, a solar simulator (AM1.5G, wavelength: 350–1800 nm, HAL-320W, Asahi Spectra Co., Ltd., Tokyo, Japan) was used as the light source. The power intensity was measured by a thermal sensor power meter (PM160T, Thorlabs, Inc., New Jersey, USA). The samples were placed on an acrylic plate, which had a hole (*Φ* 70 mm) in the middle. After emissivity calibration of each sample, the surface temperature changes of the samples during illumination of solar simulator were recorded by the infrared thermal camera.

### 4.5 Solar vapor generation and water purification experiment

In the solar vapor generation experiment, a weighing bottle (*Φ* 25 mm × 45 mm) containing 12 mL of distilled water was prepared. The samples were cut into a certain shape, as shown in Fig. 6a, and then placed on foamed styrol floating on the surface of water. The light intensity of solar simulator was adjusted as mentioned above. The mass losses of the water with different samples were monitored at intervals of 1 min using a micro analytical balance (BM-252, A&D Co., Ltd., Tokyo, Japan), which was connected to a computer. Each experiment lasted for 1 h. In the repeated seawater-evaporation experiment, artificial seawater was used instead of distilled water. Each cycle was sustained for 1 h under simulated solar light irradiation. After each cycle, the remaining seawater was removed and then replenished by unused seawater for another cycle. All of the experiments were typically carried out at an ambient temperature of 25 °C and humidity of ∼50%.

## Conflicts of interest

There are no conflicts to declare.

## Supplementary Material

NA-002-D0NA00163E-s001
