# Supplementary material for: Cellulose paper support with dual-layered nano–microstructures for enhanced plasmonic photothermal heating and solar vapor generation
Source: Nanoscale Adv. 2020 Apr 22;2(6):2339–46. doi: 10.1039/d0na00163e (PMC9417936; doi:10.1039/d0na00163e)
Supplement: NA-002-D0NA00163E-s001 [file NA-002-D0NA00163E-s001.pdf]

Electronic Supplementary Material (ESI) for Nanoscale Advances.
This journal is © The Royal Society of Chemistry 2020

Supplementary Information

Cellulose Paper Support with Dual-Layered Nano-Microstructures for Enhanced Plasmonic Photothermal Heating and Solar Vapor Generation

Yintong Huang, Yoshitaka Morishita, Kojiro Uetani, Masaya Nogi, and Hirotaka Koga*

The Institute of Scientific and Industrial Research, Osaka University, 8-1 Mihogaoka, Ibaraki, Osaka, 567-0047, Japan

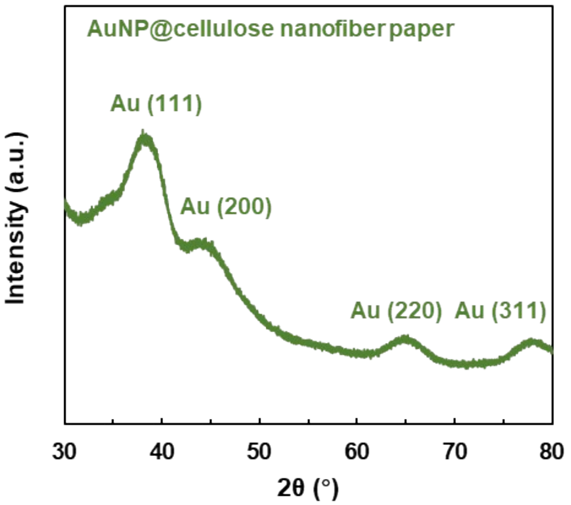

Fig. S1 X-ray diffraction pattern of the AuNP@cellulose nanofiber paper. The characteristic peaks of the Au crystal[1] at 38.2°, 44.5°, 64.7° and 77.7° are confirmed, which indicate the formation of AuNP within the cellulose nanofiber paper.

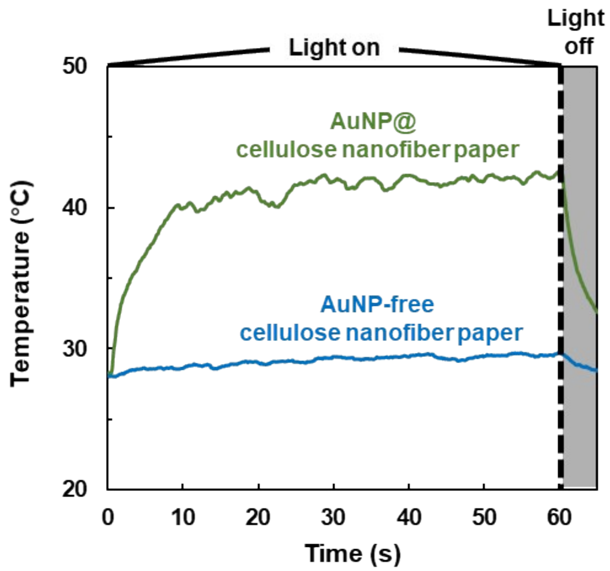

Fig. S2 Surface temperature of cellulose nanofiber paper and AuNP@cellulose nanofiber paper (AuNP content: 3.3 mmol m^{-2}) over time during surface temperature measurement under 1.0 kW m^{-2} irradiation.

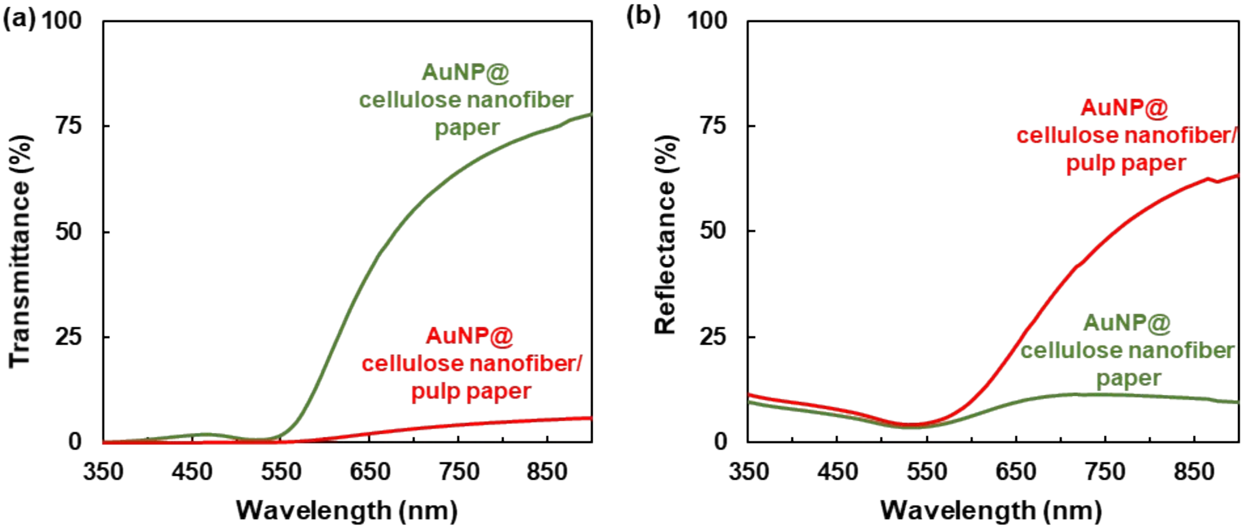

Fig. S3 UV-vis-NIR (a) transmittance spectra and (b) reflectance spectra of AuNP@cellulose nanofiber paper (thickness: ca. 100 µm, AuNP content: 3.3 mmol m^{-2}) and AuNP@cellulose nanofiber/pulp paper (thickness: ca. 520 µm, AuNP content: 3.3 mmol m^{-2}, pulp content: ca. 800 mg).

The light absorption of the AuNP@cellulose nanofiber/pulp paper, which was calculated from its transmittance and reflectance shown in Fig. S3, was higher than that of the AuNP@cellulose nanofiber paper (Fig. 4d). This result indicated that the pulp layer contributed to the enhanced light absorption. While the AuNP@cellulose nanofiber/pulp paper improved light absorption by using the pulp as a reflection layer (Fig. 4d), it showed higher reflectance than the AuNP@cellulose nanofiber paper without the pulp layer (Fig. S3b). These results suggested that a part of the reflected light by the pulp layer was absorbed by AuNPs, and the rest was still re-transmitted through the AuNP@cellulose nanofiber layer.

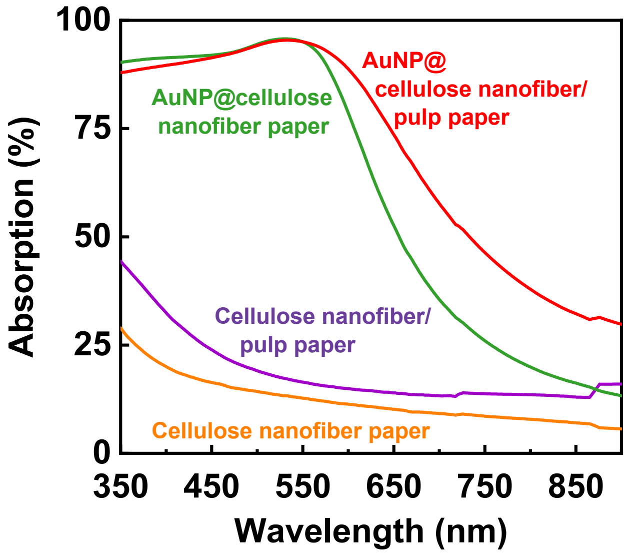

Fig. S4 UV-vis-NIR absorption spectra of AuNP@cellulose nanofiber paper (thickness: ca. 100 μm, AuNP content: 3.3 mmol m^{-2}), AuNP@cellulose nanofiber/pulp paper (thickness: ca. 520 μm, AuNP content: 3.3 mmol m^{-2}, pulp content: ca. 800 mg), cellulose nanofiber paper, and cellulose nanofiber/pulp paper (pulp content: ca. 800 mg).

The light absorption at wavelengths below 550 nm of AuNP@cellulose nanofiber/pulp paper was similar with that of AuNP@cellulose nanofiber paper, indicating the high light-absorption performance of AuNP@cellulose nanofiber paper (a top layer) in this wavelength range. On the other hand, AuNP@cellulose nanofiber/pulp paper improved light absorption in the wavelength range of 550−900 nm, as compared with AuNP@cellulose nanofiber paper. Cellulose nanofiber/pulp paper showed higher light absorption than cellulose nanofiber paper, indicating that the pulp layer (a bottom layer) showed light absorption to some extent. However, the increased light absorption of AuNP@cellulose nanofiber/pulp paper in the wavelength range of 550−900 nm was higher than the light absorption by the pulp layer. These results suggested that the pulp layer improved the light absorption efficiency of AuNPs by acting as a light-reflection layer (see also Fig. S5).

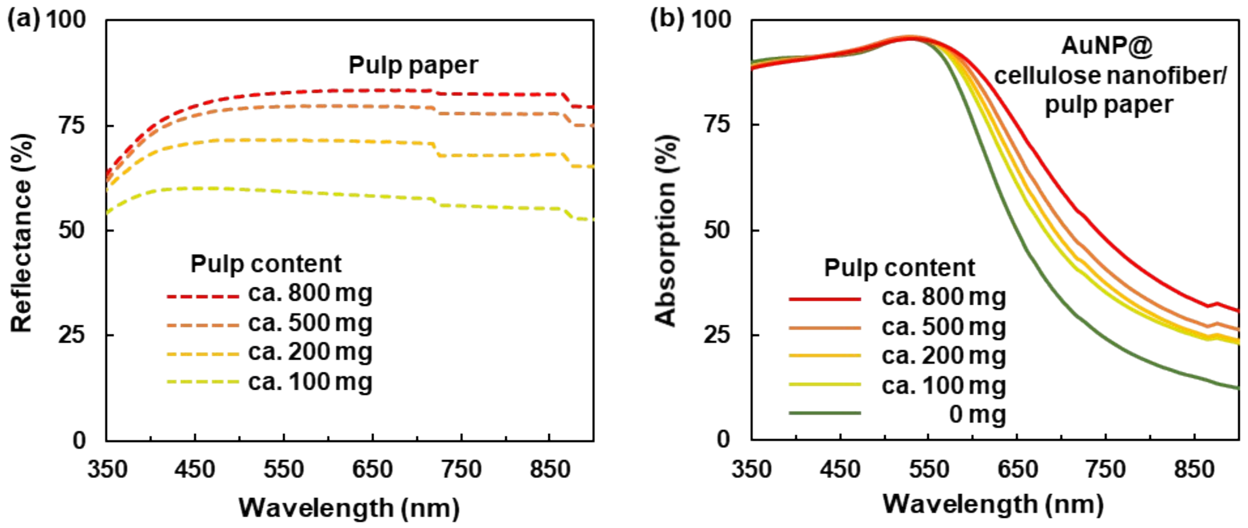

Fig. S5 (a) UV-vis-NIR reflectance spectra of pulp paper with different pulp contents (pulp content: ca. 100, 200, 500 and 800 mg, thickness: ca. 80, 130, 300 and 410 μm, respectively), and (b) absorption spectra of AuNP@cellulose nanofiber/pulp paper with different pulp contents (pulp content: 0, ca. 100, 200, 500 and 800 mg, thickness: ca. 100, 160, 210, 360 and 520 μm, respectively).

Both the reflectance of the pulp paper and the light absorption of the AuNP@cellulose nanofiber/pulp paper were increased by increasing the pulp content (thickness).

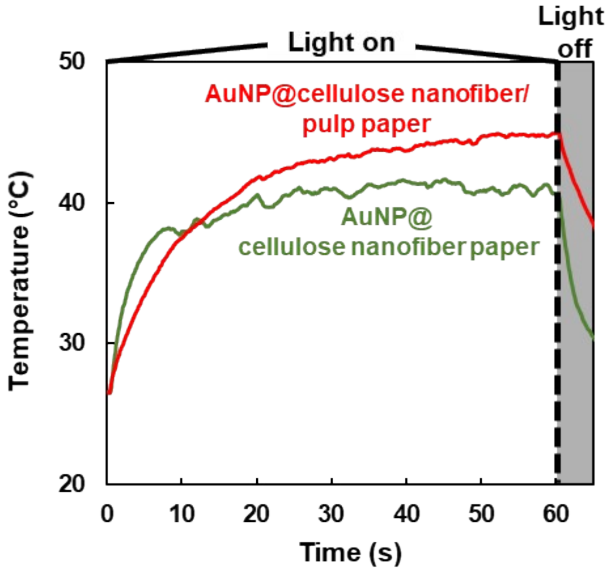

Fig. S6 Surface temperature of AuNP@cellulose nanofiber paper (thickness: ca. 100 μm, AuNP content: 3.3 mmol m^{-2}) and AuNP@cellulose nanofiber/pulp paper (thickness: ca. 520 μm, AuNP content: 3.3 mmol m^{-2}, pulp content: ca. 800 mg) over time during surface temperature measurement under 1.0 kW m^{-2} irradiation.

Reference

[1] T.Y. Suman, , S.R. Radhika Rajasree, R. Ramkumar, C. Rajthilak, and P. Perumal, The Green synthesis of gold nanoparticles using an aqueous root extract of Morinda citrifolia L. *Spectrochim. Acta - Part A Mol. Biomol. Spectrosc.*, 2014, **118**, 11–16.
